# An Unusual Case of Gastroschisis

**Published:** 2010-08-14

**Authors:** Bilal Mirza, Afzal Sheikh

**Affiliations:** Department of Pediatric Surgery, The Children's Hospital and the Institute of Child Health Lahore, Pakistan

**Keywords:** Gastroschisis, Imperforate anus, Evisceration

## Abstract

Gastroschisis is an abdominal wall defect through which intestine and rarely other organs eviscerate. It is less frequently associated with anorectal malformations. Abnormal size and shape of the defect is rarely identified in these patients. We report a case of gastroschisis with an unusual abdominal wall defect, imperforate anus and an ectopically placed vestibule. The defect was extended from right side of umbilicus to the perineum. There was evisceration of entire gastrointestinal tract (GIT), liver, gallbladder and urinary bladder. The defect was not manageable with a spring loaded silo and a sterilized blood bag was used to cover the defect. The unusual defect, associated anomalies and evisceration of unusual viscera are the main reasons for reporting the index case.

## INTRODUCTION

Gastroschisis refers to an abdominal wall defect situated usually on the right side of umbilical cord, through which intestine and rarely other abdominal viscera are eviscerated, with no covering membrane or sac. It is rarely associated with anorectal malformations [1 , 2].

The most accepted theory about the development of the defect is based on the resorption of right umbilical vein resulting in a defect on the right side of umbilical cord [1 , 3]. We report an usual case of gastroschisis in association with anorectal malformation. It may provide some insight as to the etiology of this anomaly. 

## CASE REPORT

A 24-hour-old female baby, product of spontaneous vaginal delivery, at term, in a village, weighing 2kg presented to emergency room with evisceration of intestine from abdomen. The patient was hypothermic and cyanosed. On examination baby had temperature of 96F, respiratory rate 45/min and pulse 80/min. Patient was placed under infant warmer and oxygen inhalation given via mask. Warm IV fluids infused and antibiotics started. After stabilization, the patient was shifted to the operation theatre.

The abdominal defect was about 7cm in diameter and on right side of the umbilical cord. It was extending down to the perineum. The entire GIT was eviscerated along with liver, gallbladder and urinary bladder. Spleen, ovaries and uterus were lying inside the abdominal cavity. The intestine, gallbladder and urinary bladder were matted together and a thick peel covered them in toto. Anus was absent. There was a labial prominence on right side of the defect in perineum while on left side an abnormal vestibule harbouring two openings, found. From one opening meconium was coming and from other urine passed out. There was another labial prominence lateral to the vestibule. There was no bladder or cloacal exstrophy (Fig. 1).

**Figure:**
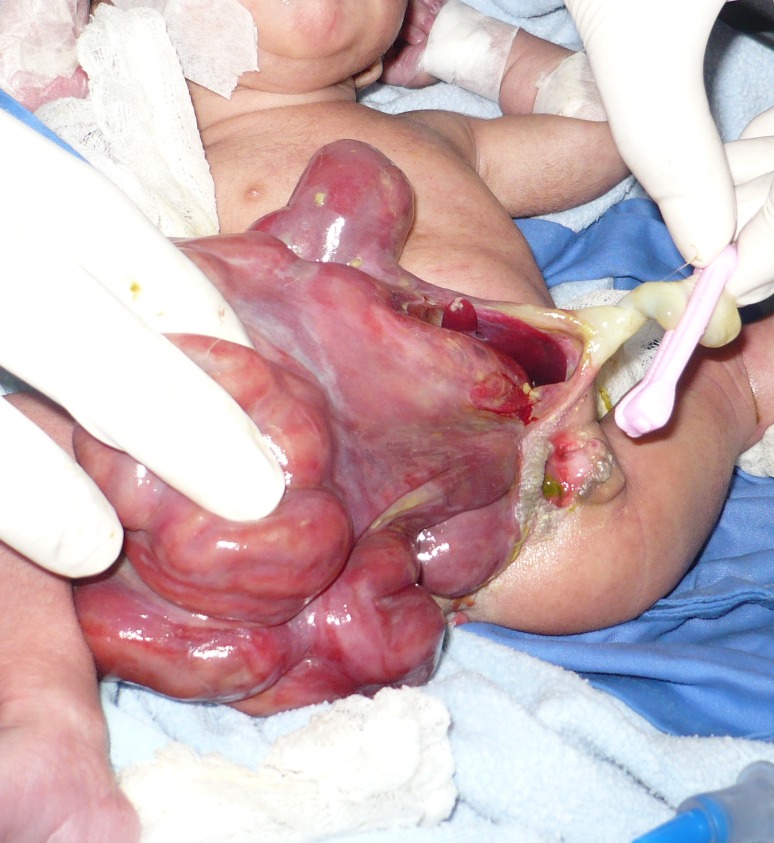
Figure 1: Defect on right side of umbilicus and extending down to the perineum; evisceration of matted and massively edematous intestinal loops, liver and gallbladder; imperforate anus and ectopically placed vestibule showing meconium inside.

It was not possible to close the defect primarily because of very limited abdominal capacity, much increased size of abdominal viscera and unusual defect. We tried spring loaded silastic silo, but it was not technically compatible with the size and shape of the defect, a sterilized blood bag was then used as silo. Post operative course remained stormy. Later baby developed septic shock and died.

## DISCUSSION

The term gastroschisis (Gastro = belly, Schisis = separation) was used, for the first time, by Taruffi in 1894, and it refers to an abdominal wall defect situated usually on the right side of umbilical cord through which mid-gut and rarely other abdominal organs eviscerate [1 , 4].

Other viscera that may eviscerate are stomach, duodenum, colon, urinary bladder, gonads, liver whole or some part of it, and gallbladder [1]. In our case the entire intestine, from stomach to rectum, was eviscerated along with liver, gallbladder and urinary bladder.

The defect in gastroschisis is usually small (&<4cm) as compared to omphalocele. The defect usually lie on the right side of umbilical cord but left sided gastroschisis has been reported in literature [1 , 5]. In our patient the defect was in usual location but extended down to the perineum.

Two most important theories regarding the development of the defect are resorption of right umbilical vein and failure of mesodermalization. According to the first theory, the defect probably resulted due to failure of the umbilical coelom to develop. The elongating intestine then has no space to expand and ruptures out in amniotic cavity. This event occurred on the right side of umbilical cord due to resorption of right umbilical vein at 4th week of gestation. According to the other, but not generally accepted theory, there is failure of mesodermalization of the anterior abdominal wall with absence of abdominal wall components [3]. In our case the defect was unusual and resorption of right umbilical vein theory cannot explain it adequately. Moreover, the existence of left sided defects, also points towards some other etiology of the defect. Following considerations can be made regarding the index case;

1. It may be a case of caudal fold defect
2. It may be a combined defect i.e. caudal fold defect and gastroschisis
3. It may be a new type of malformation
4. It may be a case of gastroschisis with unusual defect

The first two theories cannot explain the defect in absence of bladder or cloacal exstrophy which are associated with caudal fold defects. Various defect have been reported in literature and include a defect in the left hypochondrium and bilateral defects, but, none of them had associated eviscerated bowels and usual relationship with umbilical cord, as typically characterized in the gastroschisis [6 , 7]. These cases could be considered as new malformations, however, in our case the location of the defect and evisceration of abdominal content preclude its place in new malformation. The fourth option may be valid in our case. The defect, in the index case, was too long and can not result from mere resorption of right umbilical vein. This strengthens the notion of true absence of the abdominal wall that could be the result of failure of mesodermalization process. 

Gastroschisis has low association with congenital malformations as compared to the omphalocele. The reported associated anomalies are intestinal atresia, malrotation, choledochal cyst, cleft lip and palate, and sternal clefts. Its association with anorectal malformations is rarely reported [1 , 4 , 5 , 8 , 9]. In our case the anal pit was absent and an abnormal opening with meconium inside was present in ectopically placed vestibule. This might be a recto-vaginal fistula because urethral opening was lying in normal relation at ectopic site.

In our case the patient presented after 24 hours of birth with massively matted and edematous eviscerated contents. Though early cover was applied to eviscerated structure following optimization still baby went into sepsis and died. If timely referral, ideally antenatal diagnosis, had been made baby might be saved. To conclude, right umbilical vein resorption theory cannot explain every patient of gastroschisis, and failure of mesodermalization theory can be put forward to explain the unusual defect found in the reported case.

## Footnotes

**Source of Support:** Nil

**Conflict of Interest:** None declared
